# Clinical and Modifiable Factors Associated With Disability and Relapse in MOGAD: A Multicentre Cohort Study

**DOI:** 10.1002/acn3.70460

**Published:** 2026-06-23

**Authors:** Yingtao Wang, Shaoxin Tao, Qiujia Wang, Xiaoyu Xu, Hui Yao, Zheng Liu, Dawei Li, Zhandong Qiu, Jinming Han, Jiangwei Xia, Qi Wang, Jun Sun, Kai Shao, Jiao Li, Shishuang Ruan, Yi Zhong, Jun Guo, Shougang Guo, Yue Huang, Guoliang Chai, Tao Jin, Hongyu Zhou, Junwei Hao, Yinan Zhao

**Affiliations:** ^1^ Department of Neurology Xuanwu Hospital Capital Medical University, National Center for Neurological Disorders Beijing China; ^2^ Beijing Municipal Geriatric Medical Research Center Beijing China; ^3^ Key Laboratory for Neurodegenerative Diseases of Ministry of Education Beijing China; ^4^ Department of Neurology Changde Hospital, Xiangya School of Medicine, Central South University (The First People's Hospital of Changde City) Changde Hunan China; ^5^ Department of Neurology Tangdu Hospital, Fourth Military Medical University Xi'an Shaanxi China; ^6^ Department of Neurology Shandong Provincial Hospital Affiliated to Shandong First Medical University Jinan Shandong China; ^7^ Department of Neurology Henan Provincial People's Hospital Zhengzhou Henan China; ^8^ Neuroscience Center, Department of Neurology The First Hospital of Jilin University Changchun Jilin China; ^9^ Department of Neurology West China Hospital, Sichuan University Chengdu Sichuan China

**Keywords:** cohort study, modifiable risk factors, MOGAD, prognosis

## Abstract

**Background:**

Factors associated with relapse course and disability in myelin oligodendrocyte glycoprotein antibody‐associated disease (MOGAD) remain incompletely understood.

**Objectives:**

To identify clinical and modifiable factors associated with relapse and disability in MOGAD.

**Methods:**

In this ambispective multicentre cohort study using data from the Chinese Neuroimmunological Diseases (NIDBase) cohort (21 Chinese centres; January 2020–December 2024), 173 MOGAD patients with ≥ 12‐month follow‐up were analysed. Time to first relapse and time to disability, defined as Expanded Disability Status Scale (EDSS) score ≥ 2 confirmed outside acute relapse periods, were analysed using Cox regression. Nonlinear associations were assessed using restricted cubic splines.

**Results:**

Older age at onset and smoking history were associated with increased disability risk. Smoking was also associated with relapse risk, although its association was attenuated and no longer statistically significant after adjustment. BMI showed nonlinear associations with both relapse and disability outcomes.

**Conclusions:**

In MOGAD, older age at onset, smoking, and BMI may influence prognosis. These findings support further investigation of modifiable lifestyle and metabolic factors in prognostic stratification.

## 1 Introduction

Myelin oligodendrocyte glycoprotein antibody‐associated disease (MOGAD) is an autoimmune inflammatory disorder of central nervous system (CNS), characterised by the presence of serum myelin oligodendrocyte glycoprotein immunoglobulin G (MOG‐IgG) [1]. Clinically, it most commonly presents as transverse myelitis (TM), acute disseminated encephalomyelitis (ADEM) or optic neuritis (ON), and is increasingly recognised as a distinct entity among acquired demyelinating disorders [2, 3]. Although once considered rare, MOGAD is now being reported globally across all age groups, with an estimated annual incidence of 1.6–3.4 per million and a prevalence of approximately 20 per million. The increasing number of diagnoses likely reflects improved testing availability and broadened clinical awareness [1].

Compared with other CNS demyelinating disorders, MOGAD is generally associated with a more favourable prognosis, yet some patients, especially those with older age of onset, TM, or more severe attack at onset, develop severe disability [3, 4, 5, 6, 7]. Intrathecal synthesis of MOG‐IgG has also been linked with more severe attack‐related deficits [8, 9, 10, 11]. Despite growing recognition, prognosis of MOGAD remains incompletely understood.

In particular, the role of modifiable lifestyle and metabolic factors has not been well characterised. Modifiable factors such as cigarette use, obesity, and vitamin D deficiency have established associations with disease outcome in multiple sclerosis (MS) and NMOSD. However, comparable evidence in MOGAD remains limited and inconclusive [12, 13, 14]. Current evidence is limited to isolated findings, such as an association between smoking history and poorer visual recovery after a first episode of ON [13]. Most studies have instead focused on immunological, genetic, and neuroimaging biomarkers [15].

Disability in MOGAD is thought to be driven predominantly by relapse‐associated injury and incomplete recovery rather than a primary progressive process. Clarifying these relationships may improve risk stratification and inform counselling regarding potentially modifiable prognostic factors.

In this ambispective multicentre cohort study based on the Chinese neuroimmunological diseases (NIDBase) cohort, we aimed to explore associations of baseline demographic, lifestyle, metabolic and clinical factors with two clinically relevant outcomes in MOGAD: time to first relapse and time to disability. We further explored potential nonlinear associations between body mass index (BMI) and these outcomes.

## 2 Method

### 2.1 Research Design and Participants

**FIGURE 1:**
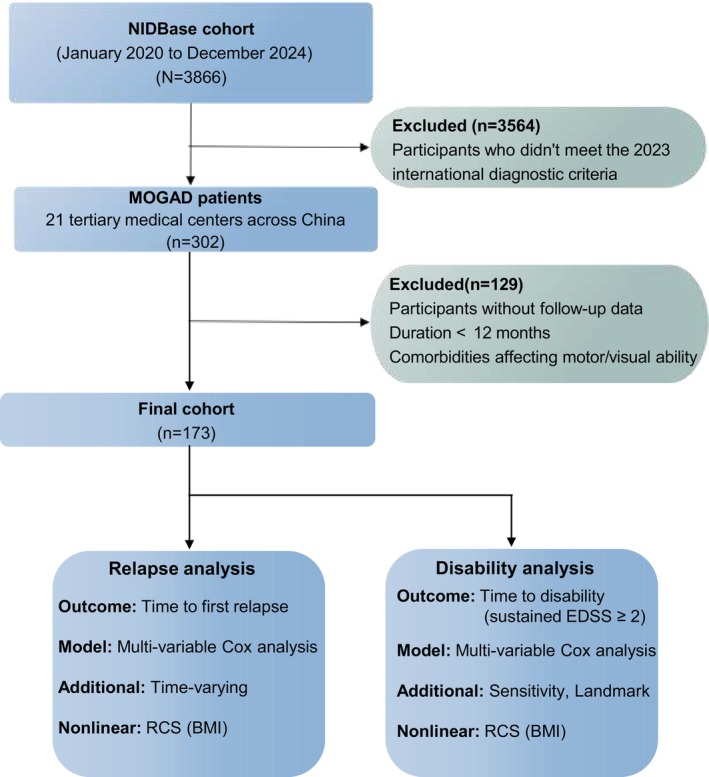
Study profile and analytical framework: flow diagram showing the enrolment of patients with MOGAD into the study cohort, application of inclusion and exclusion criteria, and final sample size for analysis. The diagram also summarises the subsequent analytical steps. MOGAD, myelin oligodendrocyte glycoprotein antibody‐associated disease.

This ambispective multicentre study used data from the NIDBase cohort which is an ongoing nationwide multicentre registry of patients with central nervous system demyelinating diseases established in January 2020. Participants are routinely followed at 3 and 6 months after enrolment and annually thereafter. Data on disease onset, initial clinical characteristics, and early relapse history were obtained retrospectively from medical records and registry documentation, whereas follow‐up outcomes after enrolment were collected prospectively. Data were extracted from the registry on 30 December 2024. The data contained 302 patients diagnosed with MOGAD according to the 2023 international diagnostic criteria, who were recruited from 21 secondary and tertiary hospitals in 15 provinces. Eligible participants met all of the following criteria: (1) fulfilment of the 2023 diagnostic criteria for MOGAD; (2) disease duration of at least 12 months; (3) clinical follow‐up of at least 12 months. Before final inclusion in the analytic cohort, medical records were reviewed for major comorbidities that could substantially confound disability assessment. Patients with severe coexisting diseases independently affecting visual or motor function, and therefore likely to interfere with EDSS evaluation or disability ascertainment, were excluded from the final analysis. In total, our analysis included 173 MOGAD patients (Figure 1). Xuanwu Hospital Capital Medical University's Ethics Committee approved the research protocol (2021‐017‐002), and written informed consent was obtained from all patients.

### 2.2 Assessment of Exposures and Covariates

We evaluated potential factors associated with outcomes in MOGAD, including demographic factors, lifestyle habits at disease onset (BMI at onset, smoking history, alcohol consumption), clinical characteristics (age at disease onset, first attack phenotype, disease duration, presence of overlap syndromes). BMI at onset was defined as the body mass index measured at, or as close as possible to, the time of the first clinical attack, based on medical records or patient recall. Baseline data were collected via systematic review of medical records, including age at onset, sex, initial attack phenotype, disease duration and presence of overlap syndromes. Additional data, such as BMI, smoking history, alcohol consumption, the geographical latitude of residence, years of education, were recorded during face‐to‐face interviews or follow‐up visits. Neurological disability was assessed using the EDSS score, based on in‐person neurological examination by trained neurologists. EDSS is a quantitative instrument for disability status assessment (ranging 0–10), with higher scores representing more severe disability [16].

Age at onset was defined as the age at first neurological symptom, and was further classified as early‐onset MOGAD (< 50 years) or late‐onset MOGAD (≥ 50 years). Educational attainment was categorised as ≤ 12 or > 12 years. BMI was calculated by dividing weight in kilograms by height in metres squared. Smoking history was defined as a binary variable (ever vs. never), where ‘ever’ referred to any prior or current use of tobacco. Alcohol consumption was defined as a binary variable (ever vs. never), where ‘ever’ referred to any prior or current alcohol use, without assessment of frequency or amount. Immunosuppressive therapy was defined as any recorded use of relapse‐prevention immunosuppressive agents during the disease course or follow‐up, excluding acute‐phase corticosteroid treatment.

### 2.3 Outcome

Two time‐to‐event outcomes were defined. The first was time to first relapse, defined as the interval from disease onset to the first relapse. The second outcome was time to disability, defined as the interval from disease onset to the first occurrence of disability, with censoring at the last follow‐up for patients who did not develop disability. Disability was defined as EDSS ≥ 2 recorded outside acute relapse periods, based on neurological assessment performed at least 1 month after relapse recovery. For landmark analysis, among patients with relapsing course, follow‐up was recalculated from the first relapse to disability, with censoring at the last follow‐up.

### 2.4 MOG‐IgG Detection

MOG‐IgG was assessed through a fixed‐cell‐based indirect immunofluorescence staining method. Two senior laboratory staff members independently evaluated the fluorescence images to ensure specific staining [17].

### 2.5 Statistical Analysis

Baseline features were shown as numbers (percentages) for categorical variables and as medians (interquartile ranges, IQR) for continuous variables with a skewed distribution. Chi‐square or Fisher's exact tests, as appropriate, were used for categorical variables, and Wilcoxon rank‐sum tests were used for continuous variables, with missing data details for each variable available in Table S1. Table S1 shows missing data in the initially identified MOGAD cohort before final analytic cohort selection. For analyses in this study, only patients with non‐missing values for the variable under analysis were included. No imputation was performed. Age at onset, geographical latitude of residence, and BMI were analysed as continuous variables in regression models; categorical cut‐offs were used only for descriptive Kaplan–Meier analyses.

Time‐to‐event outcomes were analysed using Cox proportional hazards regression. Univariable Cox models were first fitted for each candidate baseline variable. Variables (*p* < 0.05) associated with the outcome in univariable analyses were entered into multivariable models. Hazard ratios (HRs) and 95% confidence intervals (CIs) were reported.

For the analysis of first relapse, the outcome was time from disease onset to first relapse. For the analysis of disability, the outcome was time from disease onset to disability, with censoring at the last follow‐up. Disability was defined as EDSS ≥ 2 confirmed outside acute relapse periods, based on assessments performed at least 1 month after relapse recovery. A sensitivity analysis restricted to patients with relapsing MOGAD was performed to assess the robustness of the disability model. To evaluate the impact of disease activity on disability risk, a landmark analysis was performed using the first relapse as the landmark time. Among patients who experienced a relapse, follow‐up was recalculated from the time of first relapse to disability, with censoring at the last follow‐up. Patients who developed disability before the landmark time were excluded. In this framework, the time from disease onset to first relapse was considered a proxy for disease activity.

Kaplan–Meier curves with log‐rank tests were used to visualise unadjusted disability‐free survival according to selected categorical variables.

The proportional hazards assumption was assessed using Schoenfeld residuals. Although the global test suggested some deviation from proportional hazards, no individual covariate showed statistically significant evidence of non‐proportionality, and no substantial time‐dependent patterns were observed on visual inspection. Therefore, the primary Cox models were retained.

To explore potential nonlinear associations between continuous variables and time‐to‐event outcomes, restricted cubic spline (RCS) models were fitted within the Cox proportional hazards framework. The number of knots (3–5) was selected based on the Akaike information criterion (AIC), with the model yielding the lowest AIC considered optimal. When nonlinearity was detected, a threshold effect analysis was conducted using a two‐piece Cox regression model. The inflexion point was identified as the value yielding the lowest AIC.

A two‐sided *p* value below 0.05 was used to define statistical significance. All analyses were performed with R software (version 4.4).

## 3 Results

### 3.1 Baseline Characteristics

This study included 173 patients in this analysis (Figure 1). Of the 173 patients, 94 (54.3%) were female, with a median age at disease onset of 32.0 years (IQR 21.0–43.0). Overall, 128 (74.0%) had a final EDSS score < 2, while 45 (26.0%) had a score ≥ 2. Compared with patients with final EDSS < 2, those with EDSS ≥ 2 were older at onset, had fewer years of education and longer disease duration (Table 1). No between‐group differences were observed in sex, BMI, residential latitude, smoking or drinking history, onset phenotype, overlap syndrome or immunosuppressive therapy.

**TABLE 1:** Demographics and clinical information of participants.

Characteristic	Overall (*n* = 173)	EDSS < 2 (*n* = 128)	EDSS ≥ 2 (*n* = 45)	*p*
Age at onset, median (IQR)	32.0 (21.0–43.0)	31.0 (19.5–40.0)	43.0 (27.5–54.0)	0.004
Sex				
Female	94 (54.3%)	69 (53.9%)	25 (55.6%)	0.986
Male	79 (45.7%)	59 (46.1%)	20 (44.4%)	
BMI, median (IQR)	23.4 (20.5–25.8)	23.4 (20.5–25.5)	23.8 (20.3–26.2)	0.947
Smoking history				
No	84 (76.4%)	67 (79.8%)	17 (65.4%)	0.214
Yes	26 (23.6%)	17 (20.2%)	9 (34.6%)	
Drinking history				
No	95 (86.4%)	75 (89.3%)	20 (76.9%)	0.201
Yes	15 (13.6%)	9 (10.7%)	6 (23.1%)	
Educational years, median (IQR)	12.0 (9.0–16.0)	15.0 (9.0–16.0)	12.0 (6.0–14.25)	0.015
Residence latitude (°), median (IQR)	39.4 (30.8–39.9)	39.6 (30.9–40.0)	38.9 (30.8–39.9)	0.526
Duration (months), median (IQR)	41.1 (23.4–70.0)	39.8 (23.6–64.8)	50.1 (27.7–91.4)	0.041
Phenotype				
ON	42 (25.3%)	32 (25.8%)	10 (23.3%)	0.073
TM	40 (24.1%)	25 (20.2%)	15 (34.9%)	
Brain involvement	49 (29.5%)	42 (33.9%)	7 (16.3%)	
Mixed phenotype	35 (21.1%)	25 (20.2%)	11 (25.6%)	
Overlap syndrome				
No	161 (93.1%)	118 (92.2%)	43 (95.6%)	0.672
Yes	12 (6.9%)	10 (7.8%)	2 (4.4%)	
Immunosuppressive therapy				
No	69 (43.9%)	48 (42.1%)	21 (48.8%)	0.564
Yes	88 (56.1%)	66 (57.9%)	22 (51.2%)	

*Note:* Data are presented as *n* (%) or median (IQR). Missing‐data patterns in the initially identified cohort are shown in Table S1.

Abbreviations: BMI, body mass index; brain involvement, cerebral, brainstem, or ADEM‐like/encephalitic presentation at onset; EDSS, Expanded Disability Status Scale; IQR, interquartile range; mixed phenotype, two or more clinical phenotypes present simultaneously at onset; ON, optic neuritis; TM, transverse myelitis.

### 3.2 Risk of First Relapse After Disease Onset

Among patients with at least 1 year of follow‐up, 107 patients (61.8%) experienced at least one relapse. The median time from disease onset to first relapse was 8.0 months (IQR 2.8–21.9).

In univariable Cox regression analysis, male sex (HR 1.502; 95% CI 1.022–2.206; *p* = 0.038), smoking history (HR 2.008; 95% CI 1.206–3.344; *p* = 0.007), drinking history (HR 1.998; 95% CI 1.107–3.608; *p* = 0.022), and higher BMI (HR 1.095 per 1 kg/m^2^ increase; 95% CI 1.034–1.159; *p* = 0.002) were associated with an increased risk of relapse. No significant associations were observed for age at onset, educational attainment, overlap syndrome, latitude of residence or onset phenotype (Table S2).

After excluding patients with missing covariate data, 97 patients were included in the multivariable Cox model. In multivariable analysis, higher BMI remained associated with an increased risk of relapse (HR 1.131 per 1 kg/m^2^ increase, 95% CI 1.058–1.210, *p* < 0.001), whereas the associations for sex, smoking history, and drinking history were attenuated and no longer statistically significant (Table 2).

**TABLE 2:** Multivariable Cox regression analysis of factors associated with first relapse after disease onset in patients with MOGAD.

Variable	Hazard ratio (HR)	95% CI	*p*
Male (reference female)	0.928	0.486–1.773	0.822
Smoking history (reference no smoking history)	1.569	0.734–3.354	0.245
Drinking history (reference no drinking history)	1.689	0.780–3.655	0.184
BMI (per unit increase)	1.131	1.058–1.210	< 0.001

*Note:* Data are hazard ratios (HRs) with 95% confidence intervals (CIs) derived from a multivariable Cox proportional hazards model including sex, smoking history, drinking history, and BMI.

Abbreviations: BMI, body mass index; HR, hazard ratio; MOGAD, myelin oligodendrocyte glycoprotein antibody–associated disease.

The proportional hazards assumption was assessed using Schoenfeld residuals, with evidence of deviation observed at the global level. Although the global test suggested some deviation from proportional hazards, no individual covariate showed statistically significant evidence of non‐proportionality (Table S3). Visual inspection of Schoenfeld residual plots did not indicate substantial time‐dependent patterns (Figure S3). Given that BMI was the only variable that remained independently associated with relapse risk in the multivariable model, an additional sensitivity analysis was performed using a time‐varying Cox model incorporating an interaction between BMI and log(time). The time‐varying coefficient was not statistically significant (HR 0.980, 95% CI 0.923–1.041; *p* = 0.515), indicating no evidence that the effect of BMI changed over time and supporting the robustness of the primary findings (Table S4).

### 3.3 Survival Analysis for Factors Associated With Disability

Time‐to‐event was calculated from disease onset to disability or last follow‐up. Over a median follow‐up of 41.1 months (IQR 23.4–70.0), 45 patients (26.0%) reached disability, defined as EDSS ≥ 2 confirmed outside acute relapse periods, based on assessments performed at least 1 month after relapse recovery.

In univariable Cox regression analysis, older age at onset, smoking history, and TM at onset were associated with an increased risk of disability, whereas BMI, residential latitude, alcohol consumption, ON, brain involvement, and mixed phenotype at onset were not. Higher educational attainment was associated with a reduced risk of disability; however, it was not included in subsequent multivariable models due to a high proportion of missing data (Table S5).

After exclusion of patients with missing covariate data, 97 patients were included in the multivariable model. Older age at onset (HR 1.044; 95% CI 1.012–1.076; *p* = 0.006), smoking history (HR 2.570; 95% CI 1.054–6.267; *p =* 0.038) remained significantly associated with increased disability risk. The association between TM at onset and disability risk was attenuated and no longer statistically significant after adjustment (HR 2.145, 95% CI 0.938–4.908; *p* = 0.071) (Table 3).

**TABLE 3:** Multivariable Cox regression analyses of factors associated with disability (primary and sensitivity analyses) in patients with MOGAD.

Variable	Primary analysis (*n* = 97)			Sensitivity analysis (*n* = 61)		
	Hazard ratio (HR)	95% CI	*p*	Hazard ratio (HR)	95% CI	*p*
Age at onset (per year increase)	1.044	1.012–1.076	0.006	1.038	1.002–1.076	0.040
Smoking history (reference no smoking history)	2.570	1.054–6.267	0.038	3.262	1.130–9.416	0.029
TM at onset (reference no TM)	2.145	0.938–4.908	0.071	1.988	0.761–5.193	0.160

*Note:* Data are hazard ratios (HRs) with 95% confidence intervals (CIs) derived from multivariable Cox proportional hazards models. The primary analysis included all patients with complete covariate data (*n* = 97, events = 24), while the sensitivity analysis was restricted to patients with relapsing MOGAD (*n* = 61, events = 18). HR represents the relative hazard of disability per unit increase (for continuous variables) or relative to the reference category (for categorical variables).

Abbreviation: TM, transverse myelitis.

To assess the robustness of these findings in relapsing disease, a sensitivity analysis restricted to relapsing MOGAD was performed. Among 61 patients with relapsing disease, 18 disability events were observed. In the multivariable Cox model, older age at onset (HR 1.038, 95% CI 1.002–1.076; *p* = 0.040) and smoking history (HR 3.262, 95% CI 1.130–9.416; *p* = 0.029) remained significantly associated with an increased risk of disability. TM did not reach statistical significance (HR 1.988, 95% CI 0.761–5.193; *p* = 0.160) (Table 3).

To further characterise early disease activity, time to first relapse was considered as a proxy measure of relapse propensity. A landmark analysis was therefore performed from the time of first relapse to evaluate its association with subsequent disability. A total of 61 patients were included in the multivariable model, with 18 disability events observed during follow‐up. In this analysis, older age at onset remained significantly associated with an increased risk of disability (HR 1.052, 95% CI 1.013–1.093; *p* = 0.008), whereas time to first relapse, smoking history and TM were not statistically significant (Table 4).

**TABLE 4:** Multivariable Cox regression analysis of disability risk after first relapse.

Variable	Hazard ratio (HR)	95% CI	*p*
Age at onset (per year increase)	1.052	1.013–1.093	0.008
Time to first relapse (per month)	1.010	0.998–1.023	0.103
Smoking history (reference no smoking history)	2.060	0.695–6.104	0.192
TM at onset (reference no TM)	2.076	0.770–5.595	0.149

*Note:* Data are hazard ratios (HRs) with 95% confidence intervals (CIs) derived from a multivariable Cox proportional hazards model using a landmark approach, with follow‐up starting from the first relapse. HR represents the relative hazard of disability per unit increase (for continuous variables) or relative to the reference category (for categorical variables).

Abbreviation: TM, transverse myelitis.

**FIGURE 2:**
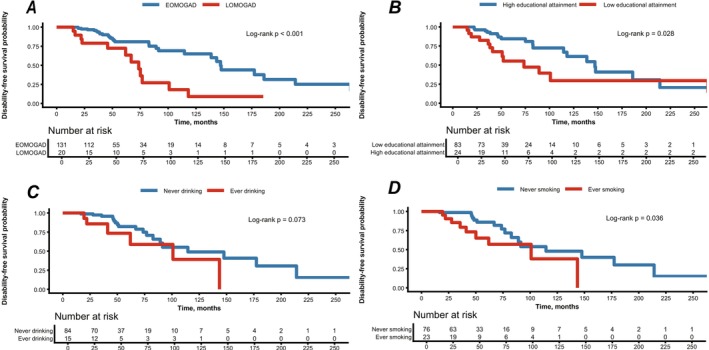
Kaplan–Meier analyses of disability outcomes according to key risk factors (A) Patients with late‐onset MOGAD (LO‐MOGAD, age of onset ≥ 50 years) had a higher probability of disability compared with early‐onset MOGAD (EO‐MOGAD age of onset < 50 years) (*p* < 0.001). (B) Patients with low educational attainment (≤ 12 years) had a higher probability of disability compared with those with high educational attainment (> 12 years) (*p* = 0.028). (C) Patients with a history of alcohol consumption showed a trend toward a higher probability of disability compared with those without alcohol consumption (*p* = 0.073). (D) Patients with a history of smoking had a higher probability of disability compared with those without smoking history (*p* = 0.036).

Kaplan–Meier survival curves demonstrated significant differences in disability‐free survival by age at onset (≥ 50 years vs. < 50 years; log‐rank *p* < 0.001), smoking history (log‐rank *p =* 0.036), and educational attainment (years of education > 12 years vs. ≤ 12 years; log‐rank *p* = 0.028). Alcohol consumption showed only a trend toward difference (log‐rank *p* = 0.073) (Figure 2).

### 3.4 Nonlinear Association of BMI With Relapse and Disability Outcomes

Restricted cubic spline analyses revealed a consistent nonlinear association between BMI and clinical outcomes across relapse and disability models (Figures S1 and S2).

For time to first relapse, a threshold effect was identified at a BMI of 23.4 kg/m^2^. Below this level, BMI was not significantly associated with relapse risk (HR 0.946, 95% CI 0.823–1.088; *p* = 0.437), whereas above the threshold, higher BMI was associated with a markedly increased hazard of relapse (HR 1.218, 95% CI 1.129–1.314; *p* < 0.001) (Table 5).

**TABLE 5:** Analysis of BMI on risk of first relapse using a two‐piecewise Cox proportional hazards model.

Variable	Hazard ratio (HR)	95% CI	*p*
BMI < 23.4 kg/m^2^ (per unit increase)	0.946	0.823–1.088	0.437
BMI ≥ 23.4 kg/m^2^ (per unit increase)	1.218	1.129–1.314	< 0.001
Male (reference female)	0.940	0.491–1.800	0.852
Smoking history (reference no smoking history)	1.658	0.766–3.588	0.199
Drinking history (reference no drinking history)	1.671	0.764–3.654	0.199

*Note:* Data are hazard ratios (HR) with 95% confidence intervals (CI) from multivariable Cox regression. The model included BMI (two‐piecewise), sex, smoking history, and drinking history. The inflexion point for BMI was identified at 23.4 kg/m^2^.

Abbreviations: BMI, body mass index; CI, confidence interval; HR, hazard ratio.

A similar nonlinear pattern was observed for disability outcomes. In the primary analysis, an inflexion point was identified at a BMI of 24.1 kg/m^2^. Below this threshold, higher BMI was associated with a lower risk of disability (HR 0.737, 95% CI 0.576–0.945; *p* = 0.016), whereas above the threshold, the association was positive but did not reach statistical significance (HR 1.190, 95% CI 0.977–1.448; *p* = 0.084) (Table 6).

**TABLE 6:** Analysis of BMI on risk of disability using a two‐piecewise Cox proportional hazards model.

Variable	Primary analysis (*n* = 92)			Sensitivity analysis (*n* = 61)		
	Hazard ratio (HR)	95% CI	*p*	Hazard ratio (HR)	95% CI	*p*
BMI < 24.1 kg/m^2^ (per unit increase)	0.737	0.576–0.945	0.016	0.567	0.408–0.787	< 0.001
BMI ≥ 24.1 kg/m^2^ (per unit increase)	1.190	0.977–1.448	0.084	1.385	1.110–1.728	0.004
Age at onset (per year increase)	1.047	1.013–1.082	0.006	1.042	1.002–1.084	0.037
Smoking history (reference no smoking history)	3.938	1.509–10.281	0.005	9.201	2.336–36.243	0.002
TM at onset (reference no TM)	2.619	1.063–6.454	0.036	4.087	1.241–13.459	0.021

*Note:* Data are hazard ratios (HRs) with 95% confidence intervals (CIs) derived from multivariable Cox proportional hazards models. The primary analysis included all patients with complete covariate data (*n* = 92, events = 24), while the sensitivity analysis was restricted to patients with relapsing MOGAD (*n* = 61, events = 18). HR represents the relative hazard of disability per unit increase (for continuous variables) or relative to the reference category (for categorical variables).

These findings were consistent in sensitivity analyses restricted to relapsing patients. An inflexion point was identified at a BMI of 24.1 kg/m^2^, where BMI demonstrated a significant bidirectional association, with decreased risk below the threshold (HR 0.567, 95% CI 0.408–0.787; *p* < 0.001) and increased risk above the threshold (HR 1.385, 95% CI 1.110–1.728; *p* = 0.004) (Table 6).

### 3.5 Long Disease Duration Subgroup

Fifty‐eight patients had disease duration at least 60 months. Over a median disease duration of 90.1 months (IQR 70.2–105.2), 20 patients (34.5%) reached disability, defined as EDSS ≥ 2 confirmed outside acute relapse periods, based on assessments performed at least 1 month after relapse recovery.

Within this subgroup, patients with EDSS ≥ 2 had an older age at onset compared with those without disability (median 39.5 vs. 24.0 years, *p* = 0.015). No significant differences were observed between groups with respect to sex, BMI, smoking or drinking history, clinical phenotype, or overlap syndrome (Table S6).

The distribution of disease duration did not differ significantly between groups (median 107.8 vs. 81.2 months, *p* = 0.386).

## 4 Discussion

This ambispective multicentre cohort study evaluated baseline demographic, lifestyle, metabolic, and clinical factors associated with relapse and disability in MOGAD. Three main findings emerged. First, higher BMI was associated with an increased risk of first relapse and showed a nonlinear association with both relapse and disability outcomes. Second, older age at onset was the most consistent predictor of disability across primary, sensitivity, and landmark analyses. Third, smoking history was associated with an increased hazard of disability, whereas its association with relapse was not robust after adjustment. These findings suggest that relapse activity and disability in MOGAD may be driven by partly distinct mechanisms, with some factors influencing inflammatory activity and others affecting recovery after attacks.

The nonlinear association between BMI and outcome is of particular interest. Higher BMI was associated with an increased risk of relapse above a threshold, while its relationship with disability followed a bidirectional pattern. This may reflect complex interactions between metabolic status and immune regulation. Excess consumption of nutrients has been linked to chronic low‐grade inflammation and altered immune responses, which may increase relapse susceptibility [18]. Higher BMI has been associated with increased risk of conversion to MS and greater inflammatory activity in clinically isolated syndrome [19]. Conversely, both low and high BMI have been associated with reduced brain volume, which may reflect impaired neuroplasticity and reduced capacity for recovery following inflammatory injury [20]. In this context, the observed nonlinear relationship between BMI and disability suggests that body composition may be associated with clinical outcomes. By contrast, most studies in MS have reported adverse effects of higher BMI on outcomes, suggesting potential differences in the underlying pathophysiology between MS and MOGAD. Further studies are needed to clarify the mechanisms linking metabolic status to disease course in MOGAD [21, 22].

The association between smoking and outcome in our cohort is also of interest. Smoking history was associated with worse disability outcomes in the primary and sensitivity analyses, which is consistent with previous observations in MOGAD suggesting poorer recovery among current smokers [13]. However, its association with relapse was not robust after adjustment. This pattern aligns with evidence from MS, where the association between smoking and relapse activity is inconsistent, whereas its relationship with disease progression and disability accumulation is more robust [23]. Smoking may have a greater impact on tissue damage and recovery processes than on relapse frequency. This may partly explain why smoking was more consistently associated with disability than with relapse in our study.

Older age at onset was the most consistent clinical factor associated with disability across analyses, including the landmark model. This finding is in keeping with previous work in MOGAD and other inflammatory demyelinating disorders [5, 23]. Age‐related reductions in remyelination capacity, neuroplasticity, and recovery from inflammatory injury may contribute to this association. In a disease such as MOGAD, in which disability is thought to result largely from incomplete recovery following attacks rather than a primary progressive process, age at onset may therefore be an important determinant of outcome.

Other factors should be interpreted more cautiously. Educational attainment differed in unadjusted analyses and disability‐free survival curves, which is consistent with observations in MS linking lower educational level to worse outcomes [24, 25]. Similarly, transverse myelitis at onset was associated with disability in univariable analysis, but the association was attenuated after adjustment and was not consistent across sensitivity analyses. These findings suggest that both variables may still have clinical relevance, but their independent contribution remains uncertain. Sex and drinking history were associated with relapse risk in univariable analyses, but these associations were not retained after adjustment, suggesting limited evidence for an independent effect in this cohort.

This study has several strengths. It was based on an ambispective multicentre cohort with standardised diagnostic criteria and longitudinal follow‐up. Time‐to‐event analyses allowed separate assessment of relapse and disability, and additional sensitivity and landmark analyses strengthened interpretation of the disability models. The use of restricted cubic spline modelling further allowed detection of nonlinear associations that would not have been captured by conventional linear approaches.

Several limitations should be acknowledged. First, the sample size was modest, particularly in subgroup and landmark analyses, which may have reduced power for some associations. Second, missing data led to substantial reductions in multivariable sample size, and no imputation was performed, introducing the possibility of selection bias. This may partly explain the higher proportion of patients with EDSS ≥ 2 observed in our cohort compared with previous studies reporting lower disability rates [26]. As with any observational study, residual confounding cannot be excluded and causal inference is limited. Third, our cohort was not restricted to incident cases and included a proportion of patients with established or relapsing disease who continued follow‐up at participating centres. This may have introduced selection bias related to disease duration and severity, and may partly account for differences between our findings and those reported in prior cohort studies. External validation in independent and more representative cohorts will therefore be important in future studies. Fourth, the duration of follow‐up was relatively limited. Although a subset of patients had longer follow‐up, the number of disability outcome events among these patients was limited, which constrained the statistical power of analyses based on extended follow‐up. Accordingly, our findings should be interpreted in the context of limited follow‐up rather than as reflecting long‐term outcomes. Fifth, treatment exposure was not modelled as a time‐varying factor, and the generalisability of these findings beyond this cohort remains to be established. Sixth, this cohort included only adult patients, as participating centres primarily managed adult neuroimmunological disease, and paediatric MOGAD was not represented; therefore, the generalisability of these findings to children is uncertain. Seventh, nadir EDSS at the initial attack was unavailable, preventing adjustment for acute attack severity, which may be an important determinant of functional disability. Eighth, detailed visual and other domain‐specific functional outcomes—such as visual acuity, gait function, and bowel or bladder dysfunction—were not systematically available, precluding assessment of important aspects of disability in MOGAD. These outcomes are highly relevant to patient quality of life and clinical decision‐making and are not fully captured by EDSS‐based measures. Finally, given that MOGAD is a relapse‐driven disease, the number of relapses is likely an important determinant of disability outcomes. However, the cumulative number of relapses is inherently a time‐dependent variable, and precise timing of individual relapse events during follow‐up was not consistently available in our dataset. Therefore, it could not be appropriately modelled within the current time‐to‐event framework without introducing potential bias. As such, this factor was not included in the analyses, which may have resulted in residual confounding.

In summary, this ambispective study demonstrates that higher BMI was associated with relapse risk and showed a nonlinear association with both relapse and disability, whereas older age at onset and smoking history were more consistently associated with disability. These findings provide additional insight into prognostic factors in MOGAD and suggest that both inflammatory activity and recovery‐related processes contribute to disability outcome. Further studies in larger cohorts are needed to validate these findings and clarify the mechanisms linking metabolic, lifestyle, and clinical factors to disease course.

## Author Contributions

Shaoxin Tao contributed to formal analysis, methodology, and writing – review and editing. Yingtao Wang, Qiujia Wang, Xiaoyu Xu, and Hui Yao were responsible for data curation, formal analysis, investigation, methodology, writing – original draft, and writing – review and editing. Zheng Liu, Dawei Li, Zhandong Qiu, Jinming Han, Jiangwei Xia, Qi Wang, Jun Sun, Kai Shao, Jiao Li, Shishuang Ruan, Yi Zhong, Jun Guo, Shougang Guo, and Yue Huang contributed to data collection, data curation, and writing – review and editing. Guoliang Chai contributed to the formal analysis, methodology, and writing – review and editing. Tao Jin and Hongyu Zhou contributed to methodology, resources, and writing – review and editing. Junwei Hao contributed to conceptualisation, formal analysis, investigation, project administration, supervision, and writing – review and editing. Yinan Zhao contributed to conceptualisation, formal analysis, investigation, project administration, supervision, funding acquisition, and writing – review and editing and had full responsibility for the decision to submit for publication and the integrity of the work as the guarantor. All authors reviewed and approved the final version of the manuscript.

## Funding

This work was supported by the Beijing Research Ward Excellence Program (Grant No. BRWEP2024W022010101); Xuanwu Hospital Talent Convergence Program (Grant No. HZ2025PYDTR003); National Natural Science Foundation of China (Grant No. 82571539); the Pilot Project for Public Welfare Development and Reform of Beijing‐affiliated Medical Research Institutes (Grant No. JYY2023‐7); the Chinese Institutes for Medical Research, Beijing (Grant No. CX23YZ15); the Project for Innovation and Development of Beijing Municipal Geriatric Medical Research Center (Grant No. 11000023T000002041657); Beijing Hospital Authority 'Dengfeng' Talent Training Plan (Grant No. DFL20220701); and the Young Beijing Scholars Program (Grant No. 020).

## Ethics Statement

Xuanwu Hospital Capital Medical University's Ethics Committee approved the research protocol (2021‐017‐002).

## Consent

All authors have approved the manuscript before submission, including the names and order of authors; all gave explicit consent to submit and obtained consent from the responsible authorities at the institute/organisation where the work has been carried out.

## Conflicts of Interest

The authors declare no conflicts of interest.

## Supporting information


**Table S1:** Overall characteristics and missing data of patients.
**Table S2:** Univariable Cox regression analysis of factors associated with first relapse.
**Table S3:** Test of proportional hazards assumption for multivariable Cox model of time to first relapse.
**Table S4:** Multivariable Cox regression model with time‐varying effect of BMI for first relapse.
**Table S5:** Univariable Cox regression analysis of risk factors for disability in patients with MOGAD.
**Table S6:** Clinical characteristics of patients with at least 60 months disease duration according to disability status (EDSS).
**Figure S1:** RCS analysis of BMI and first relapse risk in MOGAD patients.
**Figure S2:** RCS analysis of BMI and disability risk in MOGAD patients.
**Figure S3:** Assessment of the proportional hazards assumption using Schoenfeld residuals.

## Data Availability

The data that support the findings of this study are available from the corresponding author upon reasonable request.
