# Supplementary material for: Clinical and Modifiable Factors Associated With Disability and Relapse in MOGAD: A Multicentre Cohort Study
Source: Ann Clin Transl Neurol. 2026 Jun 23:10.1002/acn3.70460. Online ahead of print. doi: 10.1002/acn3.70460 (PMC13394838; doi:10.1002/acn3.70460)
Supplement: Supplementary file 1 — Table S1: Overall characteristics and missing data of patients. Table S2: Univariable Cox regression analysis of factors associated with first relapse. Table S3: Test of proportional hazards assumption for multivariable Cox model of time to first relapse. Table S4: Multivariable Cox regression model with time‐varying effect of BMI for first relapse. Table S5: Univariable Cox regression analysis of risk factors for disability in patients with MOGAD. Table S6: Clinical characteristics of patients with at least 60 months disease duration according to disability status (EDSS). Figure S1: RCS analysis of BMI and first relapse risk in MOGAD patients. Figure S2: RCS analysis of BMI and disability risk in MOGAD patients. Figure S3: Assessment of the proportional hazards assumption using Schoenfeld residuals. [file ACN3-9999-0-s001.docx]

**Supplementary Table S1. Overall Characteristics and Missing Data of Patients**

| **Characteristic** | **Overall**  **(n=302)** | **Missing, n (%)** |
| --- | --- | --- |
| **Age at onset, median (IQR)** | 33.0 (22.0-46.0) | 20 (6.6%) |
| **Sex** |  | 0 (0 %) |
| **Female, n (%)** | 163 (54.0%) |  |
| **Male, n (%)** | 139 (46.0%) |  |
| **BMI, median (IQR)** | 23.9 (21.2-25.9) | 45 (14.9%) |
| **Smoking history** |  | 114 (37.7%) |
| **No** | 143 (47.4%) |  |
| **Yes** | 45 (14.9%) |  |
| **Drinking history** |  | 114 (37.7%) |
| **No** | 156 (51.7%) |  |
| **Yes** | 32 (10.6%) |  |
| **Education attainment** |  | 147 (48.7%) |
| **Low (≤12y)** | 119 (39.4%) |  |
| **High (>12y)** | 37 (12.3%) |  |
| **Education years, median (IQR)** | 12.0 (9.0-16.0) | 150 (49.5%) |
| **Residence Latitude (°), median (IQR)** | 37.9 (30.6-39.9) | 65 (21.5%) |
| **Duration (months), median (IQR)** | 41.1 (22.1-70.0) | 117 (38.6%) |
| **Phenotype** |  | 50 (16.6%) |
| **ON** | 62 (20.5%) |  |
| **TM** | 61 (20.2%) |  |
| **Brain involvement** | 75 (24.8%) |  |
| **Mix phenotype** | 54 (17.9%) |  |
| **Baseline EDSS, median (IQR)** | 2.0 (1.0-3.5) | 58 (19.1%) |

**Supplementary Table S1. (continued)**

| **CSF OB** |  | 149 (49.3%) |
| --- | --- | --- |
| **Negative** | 118 (39.1%) |  |
| **Positive** | 35 (11.6%) |  |
| **Overlap syndrome** |  | 3(1.0%) |
| **No** | 287 (95.0%) |  |
| **Yes** | 12 (4.0%) |  |
| **Immunosuppressive therapy** |  | 68 (22.5%) |
| **No** | 122 (40.4%) |  |
| **Yes** | 112 (37.1%) |  |
| **Last EDSS, median (IQR)** | 0 (0-2) | 111(36.3%) |
| **IQR=interquartile range; BMI=body mass index; ON=optic neuritis; TM=transverse myelitis; Brain involvement = cerebral, brainstem, or ADEM-like/encephalitic presentation at onset; Mixed phenotype = two or more clinical phenotypes present simultaneously at onset; SD=standard deviation; EDSS=Expanded Disability Status Scale.** | | |

**Supplementary Table S2. Univariable Cox regression analysis of factors associated with first relapse**

| **Variable** | **Beta** | **HR (95% CI)** | ***p*-value** |
| --- | --- | --- | --- |
| **Age at onset (per year)** | 0.002 | 1.002 (0.990–1.015) | 0.717 |
| **Sex (male vs female)** | 0.407 | 1.502 (1.022–2.207) | 0.038 |
| **Smoking (yes vs no)** | 0.697 | 2.008 (1.206–3.344) | 0.007 |
| **Drinking (yes vs no)** | 0.692 | 1.998 (1.107–3.608) | 0.022 |
| **ON at onset (yes vs no)** | 0.048 | 1.049 (0.699–1.573) | 0.818 |
| **TM at onset (yes vs no)** | -0.177 | 0.837 (0.557–1.259) | 0.394 |
| **Brain involvement (yes vs no)** | 0.149 | 1.161 (0.777–1.733) | 0.467 |
| **Mixed involvement (yes vs no)** | -0.146 | 0.864 (0.470–1.589) | 0.639 |
| **BMI (per unit)** | 0.091 | 1.095 (1.034–1.159) | 0.002 |
| **Educational attainment (reference low education attainment≤12years)** | -0.480 | 0.619 (0.253–1.511) | 0.292 |
| **Overlap syndrome (yes vs no)** | -0.353 | 0.703 (0.307-1.607) | 0.403 |
| **Latitude (per degree)** | 0.009 | 1.009 (0.969–1.051) | 0.658 |

**Supplementary Table S3. Test of proportional hazards assumption for multivariable Cox model of time to first relapse**

| **Variable** | ***p*-value** |
| --- | --- |
| **Sex** | 0.905 |
| **Smoking history** | 0.255 |
| **Drinking history** | 0.238 |
| **BMI** | 0.468 |
| **Global test** | 0.017 |
| The proportional hazards assumption was assessed using Schoenfeld residuals. P values < 0.05 indicate violation of the proportional hazards assumption. The global test suggested potential deviation from proportionality, whereas no significant violations were observed for individual covariates. | |

**Supplementary Table S4. Multivariable Cox regression model with time-varying effect of BMI for first relapse**

| **Variable** | **Hazard ratio (HR)** | **95% CI** | ***p*-value** |
| --- | --- | --- | --- |
| **Body mass index (baseline effect)** | 1.161 | 1.047–1.286 | 0.005 |
| **Body mass index × log(time)** | 0.980 | 0.923–1.041 | 0.515 |
| **Sex (reference female)** | 0.926 | 0.485–1.766 | 0.815 |
| **Smoking history (reference no smoking)** | 1.608 | 0.750–3.447 | 0.222 |
| **Drinking history** **(reference no drinking)** | 1.695 | 0.774–3.710 | 0.187 |
| Data are hazard ratios (HRs) with 95% confidence intervals (CIs) derived from a multivariable Cox proportional hazards model incorporating a time-varying effect for body mass index (BMI), modelled as an interaction with log(time). The baseline HR for BMI represents its effect at the reference time point where log(time) = 0. A non-significant time-varying term suggests no strong evidence of time-dependent effect. This model was fitted to account for deviation from the proportional hazards assumption identified in the primary analysis. | | | |

**Supplementary Table S5. Univariable Cox regression analysis of risk factors for disability in patients with MOGAD**

| **Variable** | **Hazard ratio (HR)** | **95% CI** | ***p*-value** |
| --- | --- | --- | --- |
| **Male (reference Female)** | 1.533 | 0.825–2.846 | 0.176 |
| **Age at onset (per year increase)** | 1.038 | 1.018–1.059 | <0.001 |
| **BMI (per unit increase)** | 1.007 | 0.927–1.094 | 0.870 |
| **Smoking history (reference no smoking history)** | 2.435 | 1.032–5.745 | 0.042 |
| **Drinking history (reference no drinking history)** | 2.320 | 0.900–5.978 | 0.081 |
| **High Educational attainment (reference low educational attainment,≤12years)** | 0.445 | 0.223–0.891 | 0.022 |
| **Residence latitude (per unit increase)** | 1.023 | 0.956–1.095 | 0.504 |
| **Overlap syndrome (reference no overlap syndrome)** | 1.340 | 0.315–5.697 | 0.692 |
| **ON (reference no)** | 1.101 | 0.584–2.076 | 0.766 |
| **TM (reference no)** | 1.933 | 1.041–3.592 | 0.037 |
| **Brain involvement (reference no)** | 0.775 | 0.400-1.503 | 0.451 |
| **Mix phenotype (reference no)** | 1.245 | 0.580–2.672 | 0.575 |
| Data are hazard ratios (HR) with 95% confidence intervals (CI) from univariate Cox regression. MOGAD=myelin oligodendrocyte glycoprotein antibody–associated disease. BMI=body mass index; EDSS=Expanded Disability Status Scale; OB=oligoclonal band; ON = optic neuritis; TM = transverse myelitis; Brain involvement = cerebral, brainstem, or ADEM-like/encephalitic presentation at onset; Mixed phenotype = two or more clinical phenotypes present simultaneously at onset. | | | |

**Supplementary Table S6. Clinical characteristics of patients with at least 60 months disease duration according to disability status (EDSS <2 vs ≥2)**

| **Characteristic** | **Overall (n=58)** | | **EDSS < 2 (n=38)** | **EDSS ≥ 2 (n=20)** | ***p*-value** |
| --- | --- | --- | --- | --- | --- |
| Age at onset, median (IQR) | 30.05 (17.0-40.0) | | 24.0(16.0-35.0) | 39.5(20.3-55.5) | 0.015 |
| Sex | 38 (65.5%)  20 (34.5%) | |  |  | 1.000 |
| Female |  |  | 25 (65.8%) | 13 (65.0%) |  |
| Male |  |  | 13 (34.2%) | 7 (35.0%) |  |
| BMI, median (IQR) | 24.2 (20.1–25.9) | | 24.1 (19.2-25.7) | 24.5 (22.1-26.5) | 0.199 |
| Smoking history | |  |  |  | 1.000 |
| No | 30 (78.9%)  8 (21.1%) | | 20 (80.0%) | 10 (76.9%) |  |
| Yes |  |  | 5 (20.0%) | 3 (23.1%) |  |
| Drinking history | |  |  |  | 0.425 |
| No | 33 (86.8%)  5 (13.2%) | | 23 (92.0%) | 10 (76.9%) |  |
| Yes |  |  | 2 (8.0%) | 3 (23.1%) |  |
| Duration (months), median (IQR) | 90.1(70.2-105.2) | | 81.2 (67.4-104.8) | 107.8 (81.2-147.2) | 0.386 |
| Phenotype |  |  |  |  | 0.684 |
| ON | 17 (31.5%)  14 (25.9%)  16 (29.6%)  7 (13.0%) | | 10 (28.6%) | 7 (36.8%) |  |
| TM |  |  | 8 (22.9%) | 6 (31.6%) |  |
| Brain involvement |  |  | 12 (34.3%) | 4 (21.1%) |  |
| Mixed phenotype |  |  | 5 (14.3%) | 2 (10.5%) |  |
| Overlap syndrome | |  |  |  | 0.774 |
| No | 56 (96.6%)  2 (3.4%) | | 36 (94.7%) | 20 (100.0%) |  |
| Yes |  |  | 2 (5.3%) | 0 (0.0%) |  |
| Percentages were calculated among patients with available data for each variable. Missing data were not imputed. ON = optic neuritis; TM = transverse myelitis; Brain involvement = cerebral, brainstem, or ADEM-like/encephalitic presentation at onset; Mixed phenotype = two or more clinical phenotypes present simultaneously at onset. | | | | | |

**
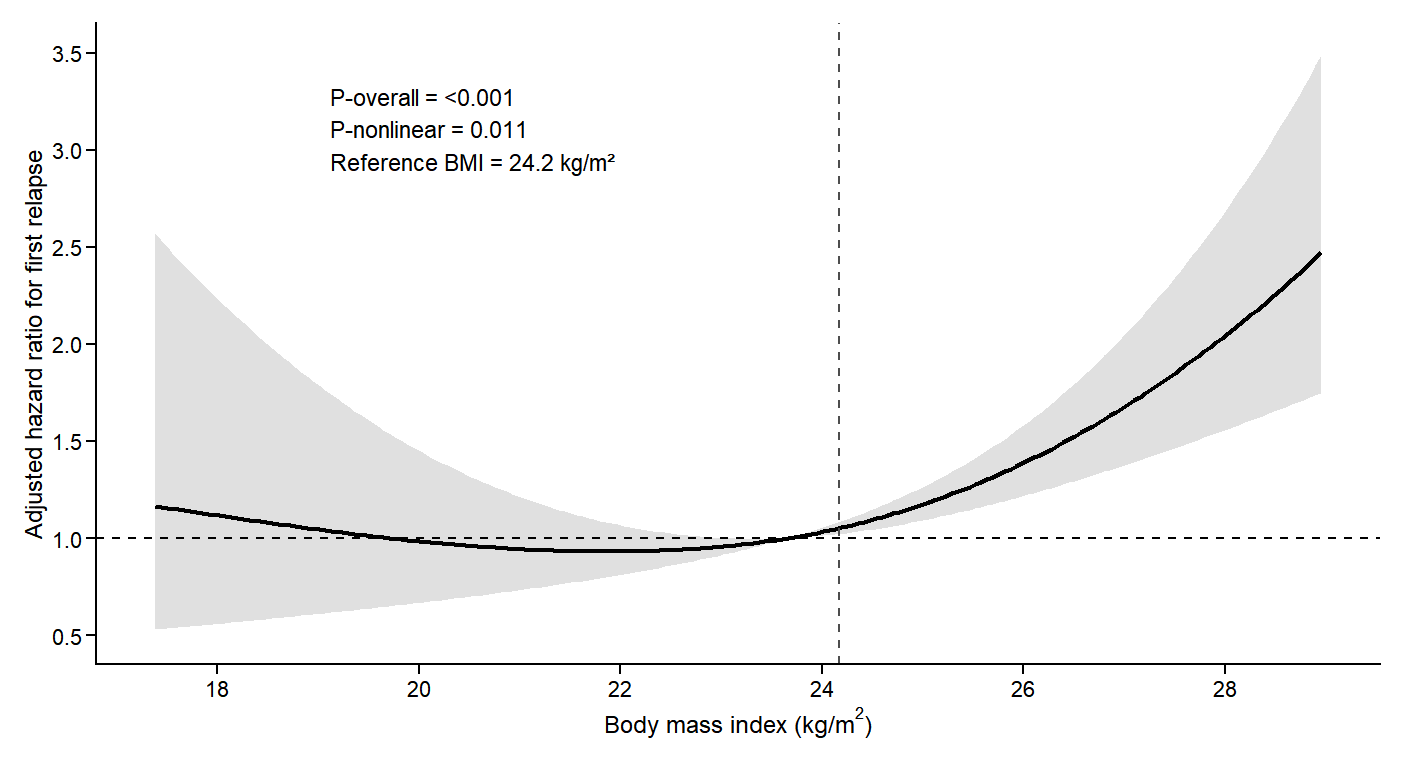
**

**Figure S1. RCS analysis of BMI and first relapse risk in MOGAD patients**

Restricted cubic spline (RCS) curves for the association between the body mass index (BMI) and first relapse risk in MOGAD patients. The black line represents the hazard ratio (HR), with HR=1 at BMI=24.2 kg/m2. The model used 3 knots and was adjusted for sex, smoking history, drinking history. P for nonlinearity=0.011. BMI=body mass index; EDSS=Expanded Disability Status Scale; HR=hazard ratio; MOGAD=myelin oligodendrocyte glycoprotein antibody-associated disease.


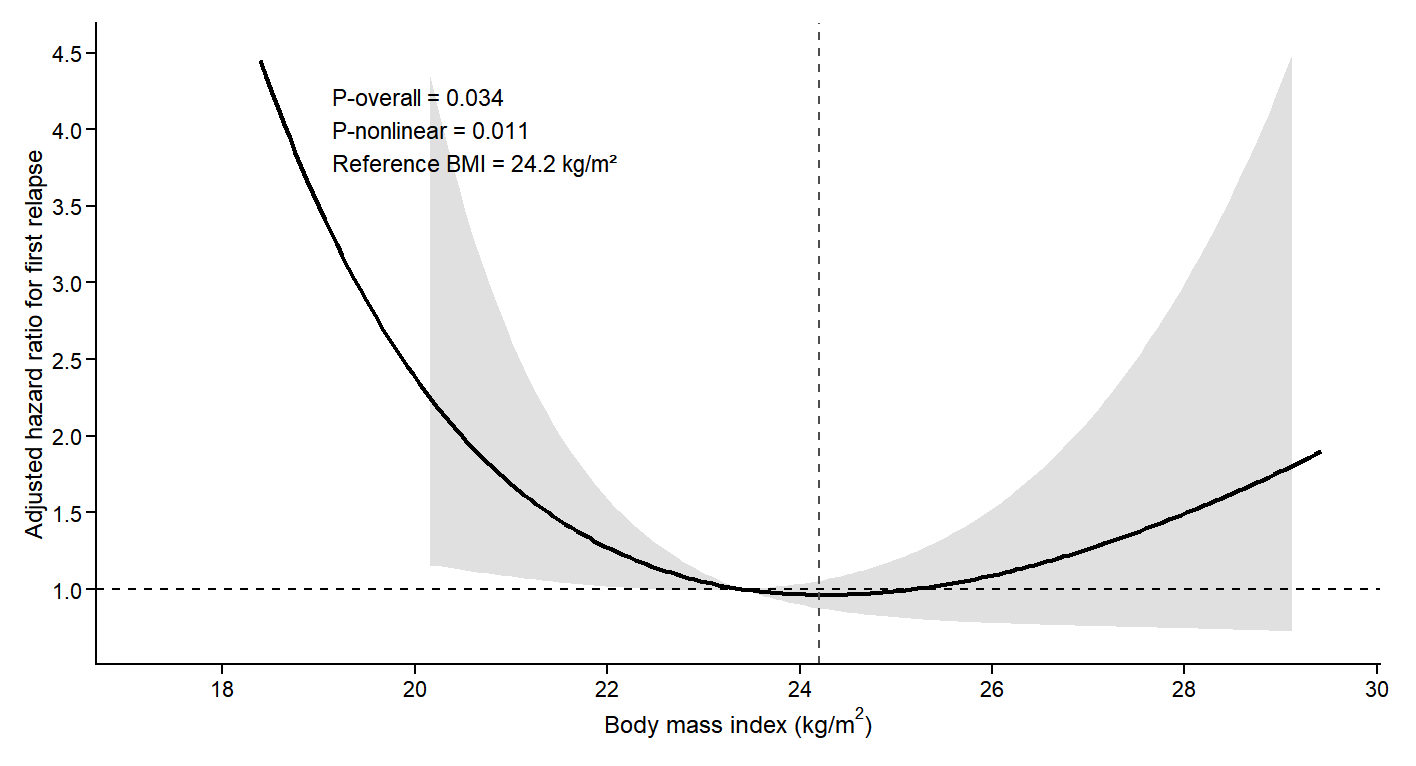


**Figure S2. RCS analysis of BMI and disability risk in MOGAD patients**

Restricted cubic spline (RCS) curves for the association between the body mass index (BMI) and disability risk in MOGAD patients. The black line represents the hazard ratio (HR), with HR=1 at BMI=24.2 kg/m2. The model used 3 knots and was adjusted for age at onset, smoking history and Transverse myelitis (TM). P for nonlinearity=0.011. BMI=body mass index; EDSS=Expanded Disability Status Scale; HR=hazard ratio; MOGAD=myelin oligodendrocyte glycoprotein antibody-associated disease.


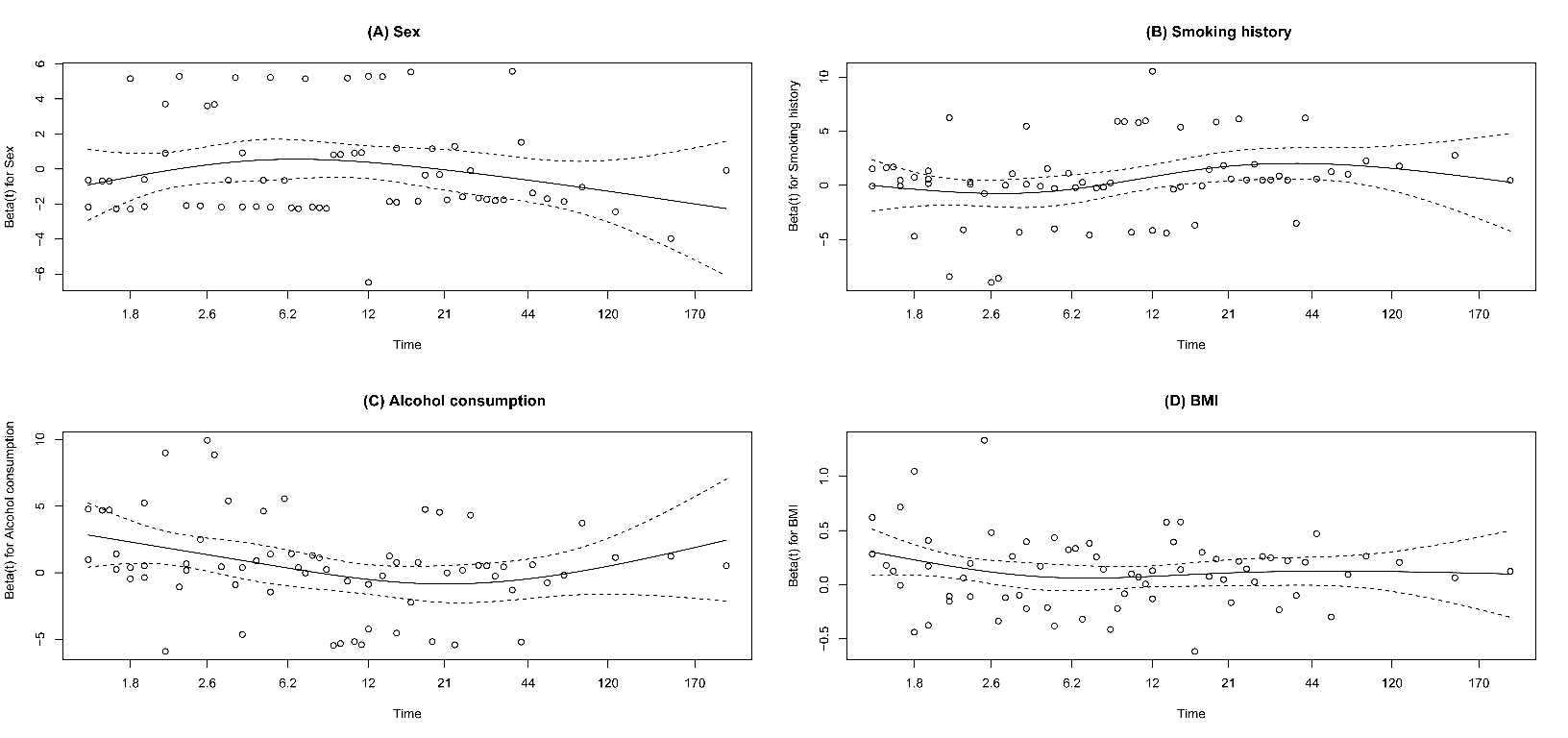


**Figure S3. Assessment of the proportional hazards assumption using Schoenfeld residuals**

Schoenfeld residual plots for key covariates in the multivariable Cox proportional hazards model for first relapse outcome: (A) sex, (B) smoking history, (C) alcohol consumption, and (D) body mass index (BMI). The solid lines represent the estimated time-varying coefficients [β(t)], and the dashed lines indicate the corresponding 95% confidence intervals. Each point corresponds to a scaled Schoenfeld residual at a given event time.

Across all variables, the estimated coefficients remained relatively stable over time, with no clear systematic trends, suggesting no strong violation of the proportional hazards assumption at the individual covariate level. Minor fluctuations were observed, particularly at later time points where the number of events was limited, resulting in wider confidence intervals.
